# Unbiased and Mobile Gait Analysis Detects Motor Impairment in Parkinson's Disease

**DOI:** 10.1371/journal.pone.0056956

**Published:** 2013-02-19

**Authors:** Jochen Klucken, Jens Barth, Patrick Kugler, Johannes Schlachetzki, Thore Henze, Franz Marxreiter, Zacharias Kohl, Ralph Steidl, Joachim Hornegger, Bjoern Eskofier, Juergen Winkler

**Affiliations:** 1 Department of Molecular Neurology, University Hospital Erlangen, Erlangen, Germany; 2 Pattern Recognition Lab, Department of Computer Science, Friedrich-Alexander-University of Erlangen-Nuremberg, Erlangen, Germany; 3 ASTRUM IT GmbH, Erlangen, Germany; Oslo University Hospital, Norway

## Abstract

Motor impairments are the prerequisite for the diagnosis in Parkinson's disease (PD). The cardinal symptoms (bradykinesia, rigor, tremor, and postural instability) are used for disease staging and assessment of progression. They serve as primary outcome measures for clinical studies aiming at symptomatic and disease modifying interventions. One major caveat of clinical scores such as the Unified Parkinson Disease Rating Scale (UPDRS) or Hoehn&Yahr (H&Y) staging is its rater and time-of-assessment dependency. Thus, we aimed to objectively and automatically classify specific stages and motor signs in PD using a mobile, biosensor based Embedded Gait Analysis using Intelligent Technology (eGaIT). eGaIT consist of accelerometers and gyroscopes attached to shoes that record motion signals during standardized gait and leg function. From sensor signals 694 features were calculated and pattern recognition algorithms were applied to classify PD, H&Y stages, and motor signs correlating to the UPDRS-III motor score in a training cohort of 50 PD patients and 42 age matched controls. Classification results were confirmed in a second independent validation cohort (42 patients, 39 controls). eGaIT was able to successfully distinguish PD patients from controls with an overall classification rate of 81%. Classification accuracy increased with higher levels of motor impairment (91% for more severely affected patients) or more advanced stages of PD (91% for H&Y III patients compared to controls), supporting the PD-specific type of analysis by eGaIT. In addition, eGaIT was able to classify different H&Y stages, or different levels of motor impairment (UPDRS-III). In conclusion, eGaIT as an unbiased, mobile, and automated assessment tool is able to identify PD patients and characterize their motor impairment. It may serve as a complementary mean for the daily clinical workup and support therapeutic decisions throughout the course of the disease.

## Introduction

Motor symptoms such as bradykinesia, rigidity, tremor, and postural instability define the diagnosis of Parkinson's disease (PD) [1]. The presence of rigidity and/or tremor defines distinct clinical phenotypes of PD [2]. Motor impairment leads to specific gait characteristic in PD, such as shuffling gait, reduced step length, impaired gait initiation, and reduced gait speed. Gait impairment and consecutively reduced mobility with loss of independency lead to the severe reduction of quality of life in PD patients [3]. The MDS Unified Parkinson Disease Rating Scale (UPDRS) - Part III is the most commonly used scale to rate motor symptoms in PD [4], and is widely accepted test to determine the efficacy of intervention in clinical studies. Disease progression is categorized using the Hoehn&Yahr (H&Y) staging [5], substantially relaying on the presence and characteristics of motor signs related to independence and quality of life. With disease progression, gait impairment increases and motor symptoms start to fluctuate, mainly recorded subjectively in patient diaries. Thus, subjective information and rating of motor signs are the basis for the daily clinician's diagnostic workup and guide therapeutic decisions.

Body-worn inertial measurement units comprising of biosensors such as accelerometers and gyroscopes are able to objectively measure changes of gait patterns. However, recent studies mostly focused on a limited number of preselected features of sensor derived signals without the use of pattern recognition approaches, or characterized individual gait abnormalities in a limited number of PD patients [6]–[11]. Our goal was to establish and validate a biosensor-based mobile and objective gait analysis system that uses pattern recognition algorithms to classify motor signs of gait impairment in PD. Pattern recognition approaches analyze several hundreds of computed biosensor derived gait signals. They are able to deliver high discriminatory power for group differentiation [12], [13]. For example, in a recent prospective study, we analyzed 2244 biomechanical movement features of 153 subjects, of which 6 developed gait impairment associated with severe patellafemoral pain syndrome. Automatic pattern recognition revealed one single feature (hip flexion-extension moment) that perfectly separated the symptomatic from the asymptomatic group [14].

In the present case-control study using a training and a validation population we combined sensor derived signals with pattern recognition approaches to test two hypotheses: Is this embedded gait analysis system using intelligent technology (eGaIT) able to assess PD-related gait changes allowing to A) separate patients from controls in order to support the clinical diagnosis of PD; and B) correctly categorize patients in different stages or with different degrees of gait impairment? This approach allows a rater and time independent, objective, and mobile assessment of gait in order to standardize staging in PD patients.

## Methods

### Standard Protocol Approvals, Registrations, and Patient Consents

Patients and controls were recruited after obtaining written informed consent. The approval from the ethical committee was received (Re.-No. 4208, 21.04.2010, IRB, Medical Faculty, Friedrich-Alexander-University, Erlangen-Nuremberg, Germany). All clinical investigations have been conducted according to the principles expressed in the Declaration of Helsinki.

### Sample and definition of clinical ratings

PD patients and controls were selected during their visit in the movement disorder outpatient center from April 2010 until February 2012 at the University Hospital Erlangen, Germany. All clinical assessment and correlation to motor signs was based on standardized consensus criteria and performed by a movement disorder specialist. PD patients were included according to the consensus criteria of the German Society of Neurology analogue to the National Institute of Neurological Disorders and Stroke (NINDS) diagnostic criteria for PD [15]. A total of 92 PD patients and 81 controls without gait impairment were enlisted in the present study, divided into two independent age matched populations, and analyzed by eGaIT in a case-control design (criterion standard) with a training and a validation group. The training population consisted of 50 patients with sporadic PD and 42 age matched controls, the validation population of 42 PD patients and 39 controls (table 1). Predominantly male PD patients were recruited. To exclude motor fluctuations between biometric gait analysis and clinical evaluation the MDS-UPDRS motor score part III [4] rating was obtained immediately (<30 min) prior to eGaIT analysis. The subitems associated with the lower extremity function (UPDRS-III_lower ex_) were defined “bradykinesia; rigidity of neck and lower extremities, tremor of lower extremities; leg agility; posture; gait; arising from chair; postural stability”. PD was staged according to H&Y [5]. To control for depression Zung Self-rating Depression Scale (SDS) was obtained [16]. PD patients reached significant higher depression scales than controls in both populations (table 1). From 81 controls, 8 were rated mildly, 2 moderately depressed, from PD patients 26 were mildly, 13 moderately, and 4 severely depressed. Exclusion criteria consisted of non-PD related gait impairments (e.g. spinal or orthopedic surgery), atypical Parkinson syndromes, spasticity, stroke, neuropathy, myelopathy, hydrocephalus, and cognitive impairment. Patients had to be able to walk independently (H&Y<4). L-Dopa equivalent dosage was calculated from all dopaminergic medication taken [17]. Motion sensor derived raw data a subgroup (27 PD patients and 16 controls) has been recently analyzed in a pilot study using different algorithms and analysis paradigms [18], [19].

**Table 1:** Characteristics of the study population.

Population:	TRAINING		VALIDATION	
Variable	PD patients (n = 50)	controls (n = 42)	PD patients (n = 42)	controls (n = 39)
**Age** (y, mean, ±SD)	63.9±10.6	60.0±11.2	65.1±9.7	60.7±11.8
**Sex** (male∶female)	36∶14	17∶25	28∶14	16∶23
**Age at onset** (y, mean, ±SD)	57.6±10.0	/	59.7±11.3	/
**Disease duration** (y, mean, ±SD)	6.5±4.7	/	5.6±4.7	/
**H&Y** (±SD)	2.1±0.9	/	2.2±0.9	/
**UPDRS-III motor score** (±SD)	18.3±11.4	0	20.7±11.8	0
**Levodopa equivalent** (mg/d, ±SD)	492±411	0	418±397	0
**Depression score** (SDS, ±SD)	49.5±13.6*	37.9±8.4	50.9±11.0*	40.2±8.0
**Weight** (kg, ±SD)	77.2±13.9	75.3±13.1	77.1±16.3	74.6±13.7
**Height** (cm, ±SD)	172±9.6	170±7.2	171±8.8	169±8.7

* : p<0.001 Student's T-test.

### Patient subgroups

For subgroup analysis, PD patients were divided into three equally sized subgroups (A) according to H&Y stages (*H&Y I, II,* or *III*), and (B) based on their UPDRS-III motor score at “best-on” state (“*UPDRS-low*”: range 0–12; “*UPRDS-mild*”: range 13–22; “*UPDRS-high*”: range 23–50). Axial affection and postural instability defining H&Y substages (1.5 or 2.5) were included (either H&Y I or II) and not categorized separately, because the major goal was to assess and classify features on gait motor function, rather than rating postural instability. Characteristics of subgroups are listed in table S1, S2.

### Standardized gait tasks

Subjects underwent *1) 10-meter walk:* Subjects passed 10 meter distance 4 times at a subjective comfortable walking speed [20]. *2) Heel-toe tapping:* While sitting, heel and toes were tapped alternately on the floor for 20 seconds requiring a flexion mainly within the ankle, comparable to the subitem “leg agility” of the UPDRS [4]. *3) Circling:* While sitting, subjects performed a sequential unilateral circling foot movement 5–10 cm above the ground floor (∼30 cm diameter) for 20 seconds to assess the movement without the influence of the body weight.

### eGaIT – Embedded Gait Analysis using Intelligent Technology

**Figure 1:**
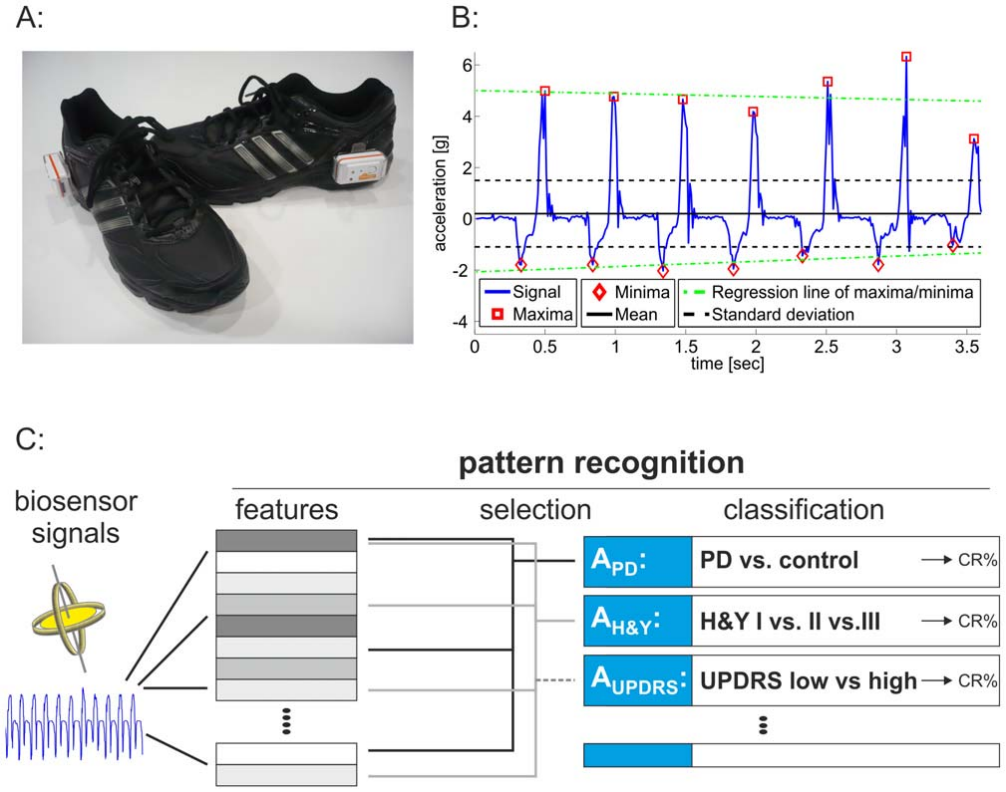
Embedded gait analysis using intelligent technology (eGaIT) concept. A: Shoe equipped with biosensors. B: Exemplary raw signal data from accelerometer with some automated computed features. C: Pattern recognition includes feature extraction from biosensor signals followed by selection, and classification of subgroups. Different pattern recognition algorithms were created: A_PD_ distinguishes between patients and controls, is generated in a training population and validated in an independent validation population. A_H&Y_ and A_UPDRS_ classify PD subgroups and include all samples.

The automated gait analysis system “*eGaIT*” consists of biosensors attached to shoes, data capture, wireless data transfer, feature extraction, and pattern recognition algorithms (figure 1, table 2,3) that performs classification experiments for each group and category (table 4) defined by the clinical rating of motor signs.

**Table 2:** Feature characteristics.

Characteristics	Sensor	Axis	Task	Total No.
**Step dependent features**	G	z-axis	Steps from 10-meter walk	10
**Step dependent features**	G/A	x-/y-/z-axis	Steps from 10-meter walk	72
**Sequence dependent features**	G/A	x-/y-/z-axis	Complete 10-meter walk sequence, 15 sec sequence from other tasks	288
**Frequency dependent features**	G/A	x-/y-/z-axis	Complete 10-meter walk sequence, 15 sec sequence from other tasks	324

Overview of individual features extracted from eGaIT based gait analysis. Feature were extracted from both shoes during defined tasks using raw sensor data from gyroscope (G) and/or accelerometer (A) using designated axes (for complete list of feature see table S3).

**Table 3:** Single features derived from pattern recognition paradigms.

Feature			Single feature analysis (PD vs. control)	
No	Name	Description	Sens.	Spec.
**10**	Minimum maximum difference	Global maximum of one step, averaged over all steps of one subject minus global minimum of one step, averaged over all steps of one subjectTask A, Gyroscope z-axis	71	44
**12**	Entropy	Uncertainty measure of the signalTask B, Accelerometer x-axis	62	72
**13**	Regression line of maxima	Regression line of all local minima and maxima in the signal sequenceTask C, Gyroscope z-axis	67	56
**16**	Variance	Measure for signal spreading, defined as the square of standard deviationTask A, Gyroscope z-axis	79	56
**17**	Root mean square	Root Mean Square or quadratic mean is a statistical measureTask A, Gyroscope z-axis	79	56
		Task B, Gyroscope z-axis	52	69
**22**	Energy in frequency band 0.5 to 3 Hz	Energy in a frequency band describes parts of distinct frequencies in the signal, typical frequency bands for specific movements can be definedTask B, Gyroscope z-axis	55	67
**24**	Windowed Energy in frequency band 0.5 to 3 Hz	Energy in frequency band of 5 second windows with an overlap of 2.5 seconds, windows from complete signal sequence are averagedTask B, Gyroscope z-axis	48	79
**26**	Power spectral density in frequency band 0.5 to 3 Hz	Energy measurement, Fourier-transform of the signals cross-correlation with itselfTask B, Gyroscope z-axis	71	51
		Task B, Accelerometer x-axis	67	69
**28**	Regression line of windowed energy	Regression line of energy values from window (2.5 s) moved through signal sequenceTask C, Gyroscope z-axis	71	56

Selected features from derived from pattern recognition algorithm “A_PD_” that show the highest difference (p<0.00001) if tested for differences between PD patients and controls (Student's T-test). Only low sensitivity and specificity is reached by single feature classification (AdaBoost). Description of feature includes the task and the sensor type/axis.

**Table 4:** Classification of PD patients and controls.

Groups and categories	n	Class. rate	Sens.	Spec.	PPV
**PD vs. CONTROL**					
**Training sample**	50∶42	**82**	**82**	**81**	**84**
**Validation sample**	42∶39	**81**	**76**	**85**	**84**
**H&Y STAGING**					
**H&Y I vs. control**	14∶39	**70**	**57**	**82**	**53**
**H&Y II vs. control**	11∶39	**86**	**91**	**82**	**59**
**H&Y III vs. control**	17∶39	**91**	**100**	**82**	**71**
**UPDRS MOTOR SCORE**					
**UPDRS-low vs. control (0–12)**	12∶39	**74**	**67**	**82**	**53**
**UPDRS-mild vs. control (13–22)**	15∶39	**81**	**80**	**82**	**63**
**UPDRS-high vs. control (23–50)**	15∶39	**91**	**100**	**82**	**68**

The pattern recognition algorithm “A_PD_” identified an optimal classifier and feature combination to reveal balanced classification rates, sensitivity, specificity, and positive predictive value (PPV) for the experiment PD patients vs. controls using the AdaBoost classifier and cross-validation in the training sample. This algorithm was validated using an independent validation sample. Features used for this algorithm include feature no.: 2, 5, 6, 8, 10, 11, 12, 13, 15, 16, 17, 18, 19, 20, 21, 22, 23, 24, 25, 26, 27, 28, 29 derived from the 10-meter walk, heel-toe tapping, and circling test (for detailed description see table S3).

### Sensor platform and setup

Inertial sensors (three axes gyroscopes and accelerometers) integrated in the SHIMMER sensor platform (Shimmer Research Ltd., Dublin, Ireland) were used. This sensor system provides an extensible platform for real-time kinematical motion recording. Sensors were attached to the lateral heel of a shoe (figure 1A). Data was captured from both feet using an accelerometer range of ±6 g (sensitivity 300 mV/g), a gyroscope range of ±500 degree/sec (sensitivity 2 mV/degree/sec), and a sampling rate of 50 Hz. Sensor signals were transmitted via Bluetooth® to a laptop computer and stored for consecutive offline analysis.

### Pattern recognition methods

Pattern recognition methods were used to train eGaIT based classification using the training population (figure 1C). Best classification algorithms were reevaluated using a second independent validation group.

#### Feature extraction

Biometric gait features were extracted from recorded gyroscope and accelerometer signals. They were obtained from single steps and gait sequences [18]. Three different kinds of features were calculated. 1) Step dependent features from single steps of the 10-meter-walk task. One feature is calculated per step and averaged over all steps of one subject, resulting in one value per subject and feature. 2) Sequence dependent features derived from the complete gait sequence in 10-meter-walk and from a 15 second period out of the other tasks. 3) Frequency features were computed from the Fourier transform of gait sequences to incorporate a frequency based analysis. Features were extracted from both shoes, which resulted in 286 features for the *10-meter walk*, and in 204 features for both the *heel-toe tapping* and the c*ircling* task, giving a total of 694 features for each subject (table 2, table S3). Exemplary accelerometer signal and typical feature during *10–meter walk* are depicted in figure 1B.

#### Feature selection and classification

The number of total features was reduced to the most relevant using Information Gain feature selection and Sequential Forward Selection. All individual features are ranked based on their information gain values [21]. Sequential Forward Selection algorithm starts with one gait feature and adds one feature after another by using the classification accuracy as an adding criterion [22]. Classifiers define decision boundaries for the separation of subgroups based on the computed features. As classifiers Linear Discriminant Analysis (LDA), AdaBoost, and Support Vector Machines (SVM) with linear and radial basis function (RBF) kernel [22] were trained and evaluated using a 10-fold cross-validation using the training group [23]. In detail, the complete training dataset was divided into 10 subgroups containing a balanced amount of patients and controls. For stratified cross-validation one subgroup was excluded from the dataset and the classifiers were trained on the remaining subjects. The resulting classification algorithms and selected features where subsequently cross-validated using the excluded subgroup. Finally, balanced classification rate, specificity, sensitivity, and positive predictive value of optimal feature sets were calculated [23]. This approach was repeated for all subgroups, resulting in an overall classification accuracy for the best classification algorithm identified. At the same time, a minimum subset of features was isolated allowing an accurate distinction between the tested subgroups.

### Statistics

Differences in clinical characteristics of patients and controls were calculated based one-way ANOVA, Pearsons's Chi-Square, or Student's T-test as indicated using the IBM SPSS Statistics software package (IBM Deutschland GmbH, Germany).

## Results

### Single feature analysis: Differences of individual gait features

**Figure 2:**
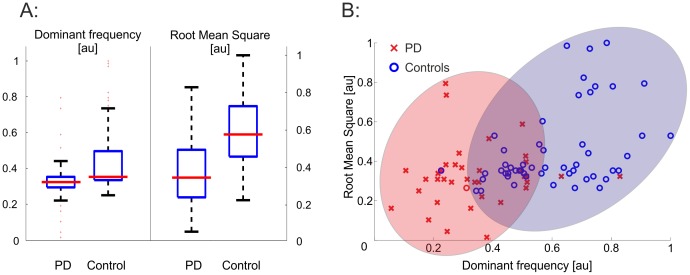
Single feature changes of gait characteristics in PD. Individual feature show significant differences between PD and controls (representative examples A, two-feature blots B), but groups overlap substantially (*: p≤0.001, T-test).

Biosensor derived raw signals from all tasks were isolated and computed into a total of 694 distinct features (figure 1, table S3). Analyzing all features individually for differences between patients and controls revealed several distinct features that significantly varied between both groups. As an example, the dominant frequency and the root mean square (potentially correlating to the walking speed and changes in alternating movements –table S3) were significantly reduced in PD patients (figure 2A). Nevertheless, a substantial overlap between PD and controls remained even if both features were plotted against each other (figure 2B). This became even clearer when looking in particular at features that were selected by pattern recognition algorithms. Here, comparing selected features individually revealed 11 feature that reached a very high significant difference (p<0.00001; Student's T-Test) between PD and control (table 3). However, only low sensitivity and specificity was achieved by AdaBoost classification using this single feature approach (table 3).

### Multi-feature analysis: Classification of PD and controls

To overcome the limits of single feature analysis, we used a pattern recognition strategy to identify cluster of features that provide specific information on biosensor based recorded gait impairments (figure 1C, table 4). The first algorithm generated by pattern recognition strategies was used to classify PD patients and controls (algorithm “A_PD_”, figure 1C). This algorithm was cross-validated using the training population. The AdaBoost classifier revealed the best classification results using a total of 64 different features (table 4, table S3). This resulted in an overall balanced classification rate of 82% for the training population.

In order to exclude overfitting by pattern recognition in a multifeature paradigm, it was important to confirm the resulting algorithm in an independent validation population (table 4). Intriguingly, the algorithm achieved similar classification results (81% correct classification accuracy, 76% sensitivity, 85% specificity, and 84% positive predictive value, table 4) supporting the hypothesis that eGaIT detects PD related gait alterations.

### Characteristics of correct and false negative classification results

Even though a validated 82% correct classification rate for the experiment “PD vs. control” already verified the applicability of eGaIT for gait analysis in PD, we also wanted to identify and characterize the remaining 18% of false classified patients. In a first attempt the classification results were divided by distinct H&Y stages (*H*&*Y I, II, III*), and different levels of motor impairment as defined by the UPDRS-III motor score (*UPDRS-low, -medium, -high*; table 4). Interestingly, more advanced H&Y stages (*H*&*Y III*) and more affected patients (*UPDRS-high*, score 23-50/108) reached very good classification results (91%), whereas classification was weaker, both at early stages (70% for *H*&*Y I*) and for mildly affected patients (74% for *UPDRS-low*). Thus, classification results improved with increased motor impairment or H&Y stages.

In a second attempt, we isolated patients from the validation group that were classified as controls by eGaIT (false negative samples). In order to define possible confounding parameters influencing the gait-based classification by eGaIT we compared the clinical characteristics between correct (n = 32) with false negative (n = 10) classified PD patients (validation sample, table 4). Notably, among the 10 false negative classified patients, the age was significantly younger than in the group of correctly classified patients (58,6 y±5.0, vs. 67.1±10.0, p = 0.001). Also, age-at-onset was younger (54,4 y±6.9, vs. 61.3±12.0, p = 0.031). Vice versa, false positive classified samples (n = 6) in the control group were older than correctly classified samples (mean age of false positive results: 67.8±10.8; compared to 59.4±11.6), without reaching statistical significance (p = 0.124). Thus, even though PD and control samples were age-matched, age in particular was the most important confounder for false classification using eGaIT.

The effect of age on false negative classification results was also observed for early H&Y stages (*H*&*Y I*) and mildly affected patients (*UDPRS-low*, table 5). In particular for the group *H*&*Y I*, age (57.5 y±3.7 vs. 66.4 y±9.9) and age-at-onset (54.5 y±3.7 vs. 64.5 y±10.5) was significantly lower in false negative classified patients. A trend, however, was observed for the *UPDRS-low* group without reaching significance (table 5 lower panel). Beneath other factors, in particular weight and gender did not differ between correct and false classified patients, thus, not supporting a confounding influence of these factors on classification quality by eGaIT.

**Table 5:** Clinical characteristics of correct and false negative classified PD patients.

Clinical characteristics	correct classified	false negative	sign. (p<0.05)
Hoehn&Yahr (H&Y I)	n = 8	n = 6	
**Sex** (male∶female)	**3∶5**	**3∶3**	
**Phenotype** (tremor-dom.∶equivalent∶akinetic-rigid)	**2∶3∶3**	**2∶1∶3**	
**Age** (y, mean, ±SD)	**66.4** (±9.9)	**57.5** (±3.7)	*
**Age at onset** (y, mean, ±SD)	**64.5** (±10.5)	**54.0** (±3.7)	*
**Disease duration** (y, mean, ±SD)	**2.0** (±1.2)	**3.9** (±3.7)	
**UPDRS motor- score** (±SD)	**12.0** (±3.3)	**8.5** (±3.6)	
**Levodopa equivalent** [17] (mg/d, ±SD)	**130** (±186)	**162** (±233)	
**Depression score** (SDS, ±SD)	**44.8** (±12.6)	**53.8** (±12.6)	
**Weight** (kg, ±SD)	**77.8** (±13.8)	**72.7** (±18.3)	
**Height** (cm, ±SD)	**172** (±11.2)	**168** (±8.8)	

UDPRS-III motor score (UPDRS-low)	n = 7	n = 5	
**Sex** (male∶female)	**4∶3**	**2∶3**	
**Phenotype** (tremor-dom.∶equivalent∶akinetic-rigid)	**2∶2∶3**	**2∶1∶2**	
**Age** (y, mean, ±SD)	**61.7** (±11.3)	**58.4** (±3.4)	
**Age at onset** (y, mean, ±SD)	**57.4** (±13.8)	**54.8** (±5.4)	
**Disease duration** (y, mean, ±SD)	**4.3** (±4.1)	**4.1** (±4.1)	
**H&Y** (±SD)	**1.6** (±0.9)	**1.1** (±0.2)	
**Levodopa equivalent** [17] (mg/d, ±SD)	**283** (±387)	**160** (±261)	
**Depression score** (SDS, ±SD)	**43.8** (±9.7)	**53.8** (±14.1)	
**Weight** (kg, ±SD)	**73.6** (±10.7)	**70.4** (±19.5)	
**Height** (cm, ±SD)	**171** (±11.5)	**166** (±8.3)	

Clinical characteristics of false negative classified PD patients at early stages (*H&Y I*) or only mild motor impairment (*UPDRS-low*; <12 UPDRS-III motor score) by the algorithm “A_PD_” compared to correctly classified patients revealed age as significantly reduced in the false negative patient groups (Student T-test, *: p<0.05).

The total UPDRS-III motor score in all false negative classified patients was slightly lower (8.5±3.6 SD, vs. 11.7±2.8 SD, p = 0.06), but not significantly different in the *H*&*Y I* group only (table 5 upper panel). Motor symptoms in early PD more frequently begin at the upper extremity and leave the lower extremity unaffected [24]. Thus, we tested, if false negative classification of PD patients depends on low or even a lack of impairment of the lower extremity in the both *H*&*Y I*, or *UPDRS-low* group. However, limiting the UPDRS-III motor score subitems of the lower extremity did not reveal differences between false negative and correctly classified PD patients (UPDRS-III_lower ex_: correct classified: 4.1±2.0 SD, vs. false negative: 3.3±2.6 SD, p = 0.49), suggesting that classification error was rather not due to the fact that false classified patients have “normal” lower extremity function.

### Classification of Hoehn&Yahr stages or different levels of motor impairment based on the UPDRS motor score

Next, we aimed to use eGaIT to distinguish patient groups at different H&Y stages (*H*&*Y I, II, III*) or with different levels of motor impairment defined by the UPDRS-III (*UPDRS-low*, -*medium*, *-high*). Since subgroups were relatively small, all samples from the training and validation population were included and classification rates were calculated using cross-validation. Thus, the aim was rather to distinguish subgroups of clinically defined patients, than support the diagnosis of PD itself.

For automated classification of Hoehn&Yahr stages a second pattern recognition algorithm was trained (“A_H&Y_”, figure 1c) and verified using cross-validation. Subgroups of patients in different H&Y stages were separated by A_H&Y_ from controls with balanced classification rates between 83% and 91% (table 6). A larger difference between H&Y stages (e.g. *H*&*Y I* vs. *H*&*Y III*) revealed the best classification results.

**Table 6:** Classification within patient subgroups.

Groups and categories	n	Classifier	Task	Features	Class. rate	Sens.	Spec.	PPV
**H&Y STAGING**								
**H&Y I vs. H&Y II**	32∶24	SVM linear (C = 15)			**83**	**75**	**91**	**86**
			A, C	8, 17, 22, 28				
**H&Y II vs. H&Y III**	24∶36	SVM linear (C = 20)			**83**	**83**	**83**	**88**
			A, B	12, 14, 18, 26, 27				
**H&Y I vs. H&Y III**	32∶36	SVM linear (C = 30)			**91**	**92**	**90**	**92**
			A, C	5, 16, 23, 24, 25				
**UPDRS MOTOR SCORE**								
**UPDRS-low vs. UPDRS-mild**	31∶30	AdaBoost (10 iterat.)			**77**	**70**	**84**	**81**
			A, B	10, 11, 12, 13, 16, 26				
**UPDRS-mild vs. UPDRS-high**	30∶31	AdaBoost (10 iterat.)			**77**	**77**	**77**	**77**
			A, B, C	8, 12, 17, 22, 24				
**UPDRS-low vs. UPDRS-high**	31∶31	SVM linear (C = 30)			**89**	**87**	**90**	**90**
			A, C	5, 17, 24, 25				

Subgroups of PD patients defined by either *H&Y I, II, III*, or UPDRS-III based levels of motor impairment (*UPDRS low*: 0–12, *mild*: 13–22; *high*: 23–50) were classified by two additional pattern recognition algorithms (“A_H&Y_, A_UDPRS_”) and cross-validated from all PD patients. Best classification results were obtained with classifier listed, resulting in the algorithms using selected features from specific tasks (A:10-meter walk, B: heel-toe tapping, C: circling).

The third algorithm (“A_UPDRS_”; figure 1C) was introduced to classify subgroups with different levels of UPDRS-III based motor impairment. Whereas differentiation between *UPDRS-low* vs. *UPDRS-mild*, or *UPRDS-mild* vs. *UPDRS-high* groups reached only 77% correct classification rates, differentiation performance of eGaIT improved substantially for the classification of *UPDRS-low* vs. *UPDRS-high* group with larger UPDRS-III score differences (89% correct classification rate, table 6). Thus, the ability to distinguish between different stages or levels of motor impairment further supports the ability of eGaIT to assess PD related gait alterations.

## Discussion

In the present study, we developed and validated eGaIT as an automated embedded gait analysis system using intelligent technology that combines multiparametric analysis using pattern recognition algorithms with unbiased biosensor derived motion data. eGaIT based classification is able to use gait alterations in PD patients corresponding to the commonly used disease stages and scores for motor symptoms in PD. In particular, eGaIT was able to A) distinguish PD patients from controls, and B) to automatically classify different disease stages and levels of motor impairment of PD patients. Therefore, eGaIT is an objective tool assessing motor symptom related gait alterations in PD, and furthermore allows automated staging and symptom monitoring in PD patients.

The usage of accelerometer based movement detection is increasing in PD. Specific motor symptoms have been characterized in PD patients by the use of biosensors. Multiple accelerometers attached to the trunk, belt, limbs or the spine are used to detect bradykinesia [25], motor-fluctuations [11], trunk stability [26], gait changes to prevent falls [9], [10], general activity [7], limb movement [8], and walking speed [27]. However, these approaches compare only single or a limited number of features derived from biosensor signals and focused primarily on specific gait characteristics of individuals or examined a very limited number of subjects. Even though the analysis of single feature correlated to specific gait symptoms [7], [8], [26], [28]–[30], the values of single feature substantially overlapped between patients from controls. Likewise, we were able to detect individual gait derived features that show significant differences between PD and control (table 2, figure 2), but due to high overlapping values only low sensitivity and specificity could be achieved (table 2). Thus, the applicability of a single feature approach is not feasible for diagnostic workup. Nevertheless, these findings clearly support the use of biosensor systems to assess motor function, but are limited in the diagnostic value in classification of PD stages.

Since single feature approaches do not sufficiently distinguish between patients and controls, we applied pattern recognition algorithms to assess the information on gait alterations concealed within the combination of a high number of biosensor signal derived features. The power of pattern recognition is increasingly appreciated in biomedicine. Modern diagnostic techniques generate a tremendous amount of information that can hardly be analyzed by simple statistical methods. Specifically developed pattern recognition paradigms using a defined set of features derived from unbiased acquired raw data are required to allow the identification of specific combination of features from several hundred features with redundant or no information (figure 1, table S3). In this respect, isolating information containing data patterns is widely used in the definition of biomarker, performing complex population based genetic analysis, and for modern imaging techniques. Pattern recognition requires a clear definition of groups or characteristics for classification. In the present study, the clinical evaluation of the examiner was used as the “gold standard” to train the algorithms. Thus, the primary goal was to recapitulate the rating by a clinician (e.g. diagnostic rating, staging, and motor performance) by eGaIT to develop this system as an objective, unbiased, and automated assessment tool supporting the clinical workup.

Definition of PD with a distinct phenotype and staging is based on the clinical rating of motor symptoms such as bradykinesia, rigidity, tremor, and postural instability. The quantitative definition of these symptoms for the clinical workup is rather broad and relies on the experience of a movement disorder specialist. Quantitative assessments such as striatal dopamine transporter imaging by single photon emission computed tomography is costly, limited available, and not suitable for disease monitoring approaches. Using the present approach, the automated movement analysis system eGaIT consists only of two sensor packages (left and right shoe). It is able to correctly classify patients from controls based on their gait alterations and motor signs with classification rates above 80%. Accuracy of the clinical diagnosis of PD ranges between 50% and 80% [31]. Even though the accuracy of eGaIT based gait analysis is higher, the clinical diagnosis includes differential diagnosis of other neurological disorders with gait impairment. Most importantly, the eGaIT based classification algorithm was confirmed by an independent validation sample (table 4). To our knowledge no other similar studies have been confirmed by an independent validation sample. Here, if multifeature analysis has been used classification results were mostly verified by cross-validation. This approach is suitable for classification of clearly defined and homogenous populations [13]. Nevertheless, even though we recruited a large patient cohort, the individual gait difference between PD patient subgroups and/or in comparison to control samples might be relatively large. This is of particular importance, because the high number of features may lead to overfitting by the classification algorithm if only one single group of patients and controls was classified. However, since the classification results for the training and validation group did not differ, we conclude that eGaIT is assessing PD related gait changes rather than individual differences of the group participants. This conclusion is further supported by the fact that with increasing levels of motor impairment or advanced stages the classification validity was improving, supporting even more a clear dependency of eGaIT analysis on PD related motor impairment.

The major advantage of the present gait analysis system relies on the objective nature of eGaIT rather than its potential ability to define the diagnosis of PD. This is in particular important for an unbiased and comparable staging of PD. Intriguingly, eGaIT was able to classify individual H&Y stages or distinct levels of motor impairment. Thus, eGaIT is able to complete and confirm the global assessment by a clinician. It is important to note that the study did not aim to detect axial involvement or postural instability (defined by H&Y substages 1.5, 2.5 or UPDRS-III subitem “postural instability”). The more detailed and better defined rating score for motor sings in PD is the MDS-UPDRS motor score [4]. It is widely used to clinically assess motor symptoms in PD, as well as one of the most important outcome measures in clinical studies for PD [32], [33]. The subjective evaluation of UPDRS-based motor symptoms by physicians, or by descriptive reports of patients restricts to a significant extent the comparability of individual assessments and validation of medical interventions. A considerable interrater variability has been reported for the UPDRS [34] and video-based teaching programs aim to improve its interrater comparability [35], [36]. In the present study, we aimed to use eGaIT as a rater-independent, objective measure of gait characteristics. We were able to correctly classify three arbitrary groups of PD patients with low, medium, and high levels of motor impairment based on the UPDRS motor score. Classification rates were even higher if UPDRS scores differed more between groups (*UPDRS-low* vs. *UPDRS-high*, table 5). This complements earlier findings, where automated movement detection of the upper limb showed the potential to correlate to a decline in motor function [6]. Previously, similar cluster analysis approaches detected changes in upper limb movement in PD patients by surface electromyography and accelerometers [30]. Furthermore, initial data from our group support the hypothesis, that combinations of upper and lower limb functional analysis might even allow a better quantification of motor impairment in PD [37].

At this point, it is also important to note, that eGaIT derived classification errors showed a strong relation to the age of the individual tested (table 4). In particular, young PD patients were more frequent misclassified as controls, or older controls were falsely classified as PD by eGaIT, respectively. Age is a very important factor for gait changes in the elderly healthy population [38]–[40]. Even though we age-matched the groups to exclude age as a confounding factor for eGaIT based analysis, the individual results emphasize that gait assessment in movement disorders substantially relies on gait characteristics in age matched control groups. Thus, future studies aiming at individual specificity and sensitivity not only have to include other diseases with gait impairments to allow differential diagnosis including atypical PD syndromes and symptomatic Parkinsonism. In addition, they also have to control for age related gait changes in the elderly healthy population in much greater detail.

In summary, our data supports the potential benefit of eGaIT as an important complementary and objective diagnostic tool for PD. In the future, it might be useful to monitor motor fluctuations in advanced stages of PD, where rapid changes in motor function occur at home during the course of a day. Similar results were obtained from accelerometers attached to limbs and the trunk of PD patients [41], including a web-based application to provide information to a clinical center [42]. Our approach is able to complement other home-based monitoring systems that currently examine distinct motor function in movement disorders of the upper limb [6], [11], [42]–[44]. Future studies are necessary including a high number of features and tasks to transfer this approach from standardized gait exercises to global gait patterns in everyday life.

## Supporting Information

Table S1 **Characteristics of the PD subgroups.** Changes between Hoehn&Yahr subgroups (*HYI-III*) were calculated by one-way ANOVA (alpha-level 0.05) (*: p<0.05; **: p≤0.001). Posthoc analysis (Bonferoni) revealed significant differences for labeled groups (p<0.05, #: in between subgroups, § compared to each other subgroup).
(DOC)

Table S2 **Characteristics of the PD subgroups.** Changes between distinct groups with different levels of motor impairment as defined by the UPDRS motor score ((*UPDRS low*: 0–12, *mild*: 13–22; *high*: 23–50) were calculated by one-way ANOVA (alpha-level 0.05) (*: p<0.05; **: p≤0.001). Posthoc analysis (Bonferoni) revealed significant differences for labeled groups (p<0.05, #: in between subgroups, § compared to each other subgroup).
(DOC)

Table S3 **List of features.** Step features extracted from gyroscope z-axis, signal sequence and frequency features applicable for all axes of accelerometer and gyroscope signals.
(DOC)
