# Supplementary material for: Unbiased and Mobile Gait Analysis Detects Motor Impairment in Parkinson's Disease
Source: PLoS One. 2013 Feb 19;8(2):e56956. doi: 10.1371/journal.pone.0056956 (PMC3576377; doi:10.1371/journal.pone.0056956)
Supplement: Table S1 — Characteristics of the PD subgroups. Changes between Hoehn&Yahr subgroups (HYI-III) were calculated by one-way ANOVA (alpha-level 0.05) (*: p<0.05; **: p≤0.001). Posthoc analysis (Bonferoni) revealed significant differences for labeled groups (p<0.05, #: in between subgroups, § compared to each other subgroup). (DOC) [file pone.0056956.s001.doc]

**Table S1: Characteristics of the PD subgroups**

| **Variable** | Hoehn&Yahr subgroups | | | |
| --- | --- | --- | --- | --- |
|  | HY I (n=32) | HY II (n=24) | HY III (n=36) | ANOVA  (a=0.05) |
| **Age** (y, mean, ±SD) | 59.7 ±11.1 # | 64.0 ±7.9 | 69.0 ±8.7 # | ** |
| **Sex** (male:female) | 19:13 | 17:7 | 28:8 |  |
| **Age at onset** (y, mean, ±SD) | 56.9 ±11.3 | 57.1 ±9.2 | 60.9 ±10.6 |  |
| **Disease duration** (y, mean, ±SD) | 3.0 ±2.8 § | 6.9 ±4.2 | 8.3 ±5.0 | ** |
| **UPDRS** motor-score (±SD) | 10.1 ±4.3 # | 19.7 ±10.9 # | 27.5 ±10.3 # | ** |
| **Levodopa equivalent** (mg/d, ±SD) | 227 ±320 # | 455 ±407 # | 667 ±363 # | ** |
| **Depression score** (SDS, ±SD) | 46.9 ±11.2 # | 47.4 ±7.4 | 53.1 ±11.9 # | * |
