# Supplementary material for: Unbiased and Mobile Gait Analysis Detects Motor Impairment in Parkinson's Disease
Source: PLoS One. 2013 Feb 19;8(2):e56956. doi: 10.1371/journal.pone.0056956 (PMC3576377; doi:10.1371/journal.pone.0056956)
Supplement: Table S2 — Characteristics of the PD subgroups. Changes between distinct groups with different levels of motor impairment as defined by the UPDRS motor score ((UPDRS low: 0–12, mild: 13–22; high: 23–50) were calculated by one-way ANOVA (alpha-level 0.05) (*: p<0.05; **: p≤0.001). Posthoc analysis (Bonferoni) revealed significant differences for labeled groups (p<0.05, #: in between subgroups, § compared to each other subgroup). (DOC) [file pone.0056956.s002.doc]

**Table S2: Characteristics of the PD subgroups**

| **Variable** | UPDRS-III subgroups | | | |
| --- | --- | --- | --- | --- |
|  | UPDRS-low  (0-12, n=31) | UPDRS-mild  (13-22, n=30) | UPDRS-high  (23-50, n=31) | ANOVA  (a=0.05) |
| **Age** (y, mean, ±SD) | 60.0 ±11.3 # | 64.5 ±8.7 | 69.2 ±8.0 # | ** |
| **Sex** (male:female) | 17:14 | 26:4 | 21:10 |  |
| **Age at onset** (y, mean, ±SD) | 54.9 ±11.2 # | 58.7 ±9.8 | 62.0 ±9.7 # | * |
| **Disease duration** (y, mean, ±SD) | 4.9 ±4.6 | 6.1 ±4.8 | 7.3 ±4.6 |  |
| **H&Y** (±SD) | 1.5 ±0.8 # | 2.0 ±0.7 # | 2.9 ±0.5 # | ** |
| **Levodopa equivalent** (mg/d, ±SD) | 353 ±413 # | 410 ±420 | 610 ±343 # | * |
| **Depression score** (SDS, ±SD) | 46.8 ±10.4 | 48.8 ±10.1 | 52.8 ±11.8 |  |
