# Supplementary material for: Unbiased and Mobile Gait Analysis Detects Motor Impairment in Parkinson's Disease
Source: PLoS One. 2013 Feb 19;8(2):e56956. doi: 10.1371/journal.pone.0056956 (PMC3576377; doi:10.1371/journal.pone.0056956)
Supplement: Table S3 — List of features. Step features extracted from gyroscope z-axis, signal sequence and frequency features applicable for all axes of accelerometer and gyroscope signals. (DOC) [file pone.0056956.s003.doc]

**Table S3: List of features**

| **Step dependent features, calculated only for z-axis of gyroscope** | | |
| --- | --- | --- |
| **No** | **Name** | **Description** |
| **1** | Step duration | Duration of the step from begin of swing phase to end of stance phase |
| **2** | Rise gradient of swing phase | Gradient from begin to maximum positive rotation of swing phase in sagittal plane |
| **3** | Rise gradient of stance phase | Gradient from begin to maximum positive rotation of stance phase in sagittal plane |
| **4** | Fall gradient of swing phase | Gradient from maximum positive rotation of swing phase to heel-on in sagittal plane |
| **5** | Fall gradient of stance phase | Gradient from maximum positive rotation of stance phase to toe-off in sagittal plane |

10 features: 2 (left and right sensor) x 5 features

| **Step dependent features, calculated for all gyroscope and accelerometer axis** | | |
| --- | --- | --- |
| **No** | **Name** | **Description** |
| **6** | Minimum | Global minimum of one step, averaged over all steps of one subject |
| **7** | Minimum standard deviation | Standard deviation of minima for all steps of one subject |
| **8** | Maximum | Global maximum of one step, averaged over all steps of one subject |
| **9** | Maximum standard deviation | Standard deviation of maxima for all steps of one subject |
| **10** | Minimum maximum difference | Feature 8 - feature 6 |
| **11** | Mean value | Mean value of one step, averaged over all steps of one subject |

72 features: 2 (left and right sensor) x 6 (sensor axis) x 6 features

| **Sequence dependent features, calculated for all gyroscope and accelerometer axis** | | |
| --- | --- | --- |
| **No** | **Name** | **Description** |
| **12** | Entropy | Uncertainty measure of the signal |
| **13** | Regression line of maxima | Regression line of all local minima and maxima in the signal sequence |
| **14** | Regression line of minima |
| **15** | Mean value | Complete signal sequence averaged |
| **16** | Variance | Measure for signal spreading, defined as the square of standard deviation |
| **17** | Root mean square | Root Mean Square or quadratic mean is a statistical measure |
| **18** | Histogram area | Summed up selected area of signals histogram |
| **19** | Integral | Expresses the area of the region in xy-plane bounded by the signal |

288 features: 3 (no of tasks) x 2 (left and right sensor) x 6 (sensor axis) x 8 features

| **Frequency dependent features, calculated for all gyroscope and accelerometer axis** | | |
| --- | --- | --- |
| **No** | **Name** | **Description** |
| **20** | Dominant frequency | Characterizes the main speed during exercise |
| **21** | Energy ratio | Complete signal sequence energy divided by energy value of dominant frequency |
| **22** | Energy in frequency band 0.5 to 3 Hz | Energy in a frequency band describes parts of distinct frequencies in the signal, typical frequency bands for specific movements can be defined |
| **23** | Energy in frequency band 3 to 8 Hz |
| **24** | Windowed Energy in frequency band 0.5 to 3 Hz | Energy in frequency band of 5 second windows with an overlap of 2.5 seconds, windows from complete signal sequence are averaged |
| **25** | Windowed Energy in frequency band 3 to 8 Hz |
| **26** | Power spectral density in frequency band 0.5 to 3 Hz | Energy measurement, Fourier-transform of the signals cross-correlation with itself |
| **27** | Power spectral density in frequency band 3 to 8 Hz |
| **28** | Regression line of windowed energy | Regression line of energy values from window (2.5 s) moved through signal sequence |

324 features: 3 (no. of tasks) x 2 (left and right sensor) x 6 (sensor axis) x 9 features
